# Analysis of Interfacial Mechanical Properties of Steel Beams Strengthened with CFRP Sheets under Temperature and Creep

**DOI:** 10.3390/polym14122384

**Published:** 2022-06-12

**Authors:** Ni Zhang, Xuetian Gu, Wenyu Hou

**Affiliations:** 1 School of Civil Engineering, Liaoning Technical University, Fuxin 123000, China; z1234567815@126.com; 2 School of Transportation Engineering, Shenyang Jianzhu University, Shenyang 110168, China; houwenyu61@163.com

**Keywords:** CFRP sheet, steel beam, temperature, creep, interface performance

## Abstract

Under the combined action of temperature and creep of CFRP (Carbon Fiber Reinforced Polymer) sheet, the interface between CFRP sheet and steel beams which are strengthened with CFRP sheet will produce relative slip. This slip will affect the interface interaction, reduce the bearing capacity and stiffness of members as well as increase the deformation. In this paper, the elastic method is used to introduce the creep effect of CFRP sheet and the temperature effect of steel beam. The calculation formulas of interface slip between CFRP sheet and steel beam, CFRP sheet tension and steel beam deformation under the combined action of temperature and CFRP creep are established. The accuracy of the analytical formula is verified by finite element analysis using the software ABAQUS. The results show that the CFRP sheet tension is smallest at the beam end while largest at the middle of the span. When the stiffness reaches about 3 ka, CFRP sheet tension basically does not change. When the temperature increases by 5 °C, the tensile force of CFRP sheet increases by about 3.7 kN, 1.8 kN and 2.3 kN, respectively. The increase of stiffness under creep has little effect on the change of CFRP sheet tension. The deformation is largest in the middle of the span while smallest at the beam end. Stiffness, temperature (5–25 °C), CFRP thickness and stiffness under creep have little effect on deformation. When the load increases by 5 kN under creep, the deformation increases by about 2.2 × 10^−7^ mm, 1.8 × 10^−6^ mm and 9.4 × 10^−7^ mm, respectively.

## 1. Introduction

Carbon fiber reinforced polymer (CFRP) has the advantages of light weight, high strength, good corrosion resistance and fatigue resistance. It has not only been widely used in aerospace, marine engineering and other fields, but has also gradually become an important new material for civil engineering. In recent years, experts and scholars have carried out extensive research on mechanical properties and practical engineering applications of externally bonded CFRP reinforced steel structures [1,2,3,4,5]. When the steel structure is strengthened with CFRP sheet, there will be relative slip between the CFRP sheet and the steel structure, which may eventually lead to the peeling failure of the interface. Previous studies [6] had shown that interface peeling failure is an important reason why CFRP sheet cannot achieve the effect of strengthening and repairing steel structures.

Many experts and scholars have studied the interfacial mechanical properties of steel structures strengthened with CFRP. Xia and Teng [7] studied the bond slip relationship between CFRP and steel through single shear specimens. The interfacial shear stress and slip were approximately calculated by recording the longitudinal strain of CFRP plate. It was found that the interfacial shear stress increases first and then decreases with the increase of slip. Based on the experimental data, a bilinear bond slip model was proposed. Wang et al. [8] comprehensively studied the bond slip relationship of CFRP/steel single shear specimen interface by using three-dimensional digital image correlation technology. Based on the test results, the key parameters of bilinear model and trilinear model were fitted so that the application range of the bond slip curve has become wider. Pang et al. [9] studied the bond properties of CFRP/steel single lap specimens under monotonic load and quasi-static cyclic load. It is found that the quasi-static cyclic load reduces the ultimate load of araldite-2015 adhesive strengthened specimens. The bond slip relationship under quasi-static cyclic loading can be traced according to the bond slip curve under monotonic loading.

The ultimate bearing capacity changes accordingly. It depends on the variation of bond strength of the CFRP/steel interface. A large number of studies showed that the interfacial bond strength increased with the increase of CFRP length [10,11,12,13,14]. However, when the bond length of CFRP exceeds a certain bond length, the bearing capacity will not continue to increase, indicating the existence of effective bond length. The bonding strength is also affected by the type of adhesive. The greater the bonding deformation ability, the higher is the interfacial bonding strength [7]. Even if the bonding adhesive is equal, the stiffness of CFRP plate also affects the bonding strength of the interface. The greater the stiffness of CFRP, the larger is the bond strength [15]. If the interfacial bond strength is strong enough to cause CFRP fracture, the bond strength depends on the performance of CFRP plates [13]. In addition to that, the thickness of the adhesive layer also affects the interfacial bonding strength. The increase of the thickness of the adhesive layer generally improves the interfacial bonding strength. However, the thicker the adhesive layer, the worse is the failure mode of the bonding interface. Different failure modes lead to different bearing capacity [11].

In theory, researchers mainly use the prediction model based on interface strength and the prediction model based on fracture mechanics to predict the strength of CFRP steel joints. The prediction model of interfacial strength [16] considers that the strength of CFRP and steel is much higher than that of the adhesive layer. Only when the maximum shear stress of the adhesive layer exceeds the limit value, the interface between CFRP and steel fails. This model can well predict the short bond length of CFRP. However, the existence of effective length and the failure mechanism of the bonding interface cannot be explained [17]. The prediction models [18,19] based on fracture mechanics require that the bond slip relationship of the interface is considered and that the bond strength is predicted in combination with the interface fracture energy. The research shows that the model based on fracture mechanics can accurately predict the bond strength of CFRP steel joints and reasonably explain the effective bond length of the interface [20].

For CFRP steel structures, the most common failure mode is interface failure mode [21]. The main influencing factors of interface failure mode are the type of CFRP, the type of adhesive, the thickness and the treatment of the steel surface. The higher the elastic modulus of CFRP, the easier it is to show the tensile failure of CFRP. The failure models of CFRP strengthened specimens with ordinary elastic modulus are mainly the interface failure between steel and rubber and the delamination between CFRP layers [22]. Compared with the interface failure between CFRP and adhesive, the interface between steel and adhesive is more prone to peeling failure. Through reasonable roughness treatment on the steel surface, the interface failure between steel and adhesive can be effectively avoided [23,24]. Fernando et al. [25] studied three common steel surface treatment methods: solvent cleaning, surface grinding and sand blasting. The results show that sand blasting surface treatment can not only improve the surface energy and surface roughness of steel, but also change the chemical composition of the steel surface. This increases the interfacial bonding strength between adhesive and steel to effectively avoid the peeling damage of the interface between CFRP and steel.

There are only some preliminary explorations on the influence of temperature on the interface properties of CFRP steel structures. Zhao et al. [26,27] found that a change of temperature would lead to change of the adhesive’s bonding properties, thus changing the stress distribution and failure mode of the interface during the tensile process of CFRP plate steel double lap specimens. Temperature alternation would reduce the bond strength between CFRP plate and steel. Wan et al. [28] analyzed the shear stress distribution at the interface of FRP steel composite beam caused by temperature difference and found that the interface shear stress at the end of a composite beam was the largest. The above research results provide a certain theoretical and experimental support for the design and application of externally bonded CFRP-steel structure fatigue reinforcement and repair technology.

In recent years, experts and scholars have analyzed and calculated the interface stress of concrete beams strengthened with CFRP. Frost et al. [29] proposed the close-form high-order calculation model, which uses concrete to complete the high-order theory and fracture mechanics theory. Based on the composite mechanics and virtual work principle, the governing equations with the displacement and interfacial shear stress of RC beam and FRP sheet as unknown parameters are established. The displacement components of RC beam, adhesive layer and FRP sheet and the stress components of each layer of fiber are determined by boundary conditions. The model can accurately calculate the interfacial bond shear stress between FRP and concrete. Malek et al. [30] assume that concrete, reinforcement, adhesive layer and FRP sheet are isotropic linear elastic materials. The FRP sheet and concrete are well bonded. The horizontal displacement of the adhesive layer and the section strain of the strengthened beam are linearly distributed along the respective section heights, and the influence of the bending moment in the concrete beam on the FRP concrete interface bond shear stress is ignored. Based on the linear elastic theory, the simplified calculation method of interface stress is derived. Smith, Teng et al. [31,32] established the differential control equation of interface stress of FRP strengthened beams under general loads on the basis of deformation coordination conditions and considering the influence of bending deformation of concrete beams and axial deformation of reinforced sheets on interface stress. The general expression for calculating the peel normal stress and the specific expression for calculating the interfacial bond shear stress of the strengthened beam under the action of uniformly distributed load, single point concentrated load and two symmetrical concentrated loads are given. Most of the above analytical studies on the bond properties between FRP and concrete are based on the strength theory, that is, when the bond shear stress of the interface reaches the bond strength, the interface will be bond damaged. Leung and Yang [33] analyzed the interface bond failure process based on the energy theory, and simulated the interface bond failure process as the interface shear crack propagation. It is considered that when the energy release rate at the crack tip reaches the interfacial shear fracture energy, the crack will expand forward. An expression of the equivalent interfacial bond strength related to interfacial fracture energy, initial residual friction stress, shear modulus of the binder and thickness of the bonding layer is derived by using crack propagation criterion based on the energy method.

From the research status, it can be seen that there are relatively many studies on the interface interaction between steel beam and FRP sheet, while the analysis of interface slip, CFRP sheet tension and steel beam deformation under the joint influence of temperature and creep is rare. When externally bonded CFRP is used to strengthen and repair steel structures, the interfacial bonding performance between CFRP and steel members is one of the decisive factors to ensure that they can work together and bear stress together. Under the combined action of temperature and creep of CFRP sheet, the interface between CFRP sheet and steel beam strengthened with CFRP sheet will produce relative slip. This slip will affect the interface interaction, reduce the bearing capacity and stiffness of members and increase the deformation. Thus, the service life of the structure is reduced. Therefore, it is of great scientific significance and engineering application value to study the interfacial properties of steel beams strengthened with CFRP under the action of temperature and creep of CFRP sheet. CFRP reinforced structures will be affected by temperature and creep during service. It is very important to study the influence of these environmental conditions on the behavior and bonding capacity of the CFRP bonding interface. Therefore, based on previous studies, this paper uses the elastic method. The creep effect of CFRP sheet and the temperature effect of steel beam are introduced, and the calculation formulas of interface slip between CFRP sheet and steel beam, CFRP sheet tension as well as steel beam deformation under the combined action of temperature and CFRP creep are given. The calculation formulas under different loads are given, and the effects of different design parameters are analyzed.

## 2. Interface Slip Analysis

### 2.1. Interface Slip Calculation

**Figure 1:**
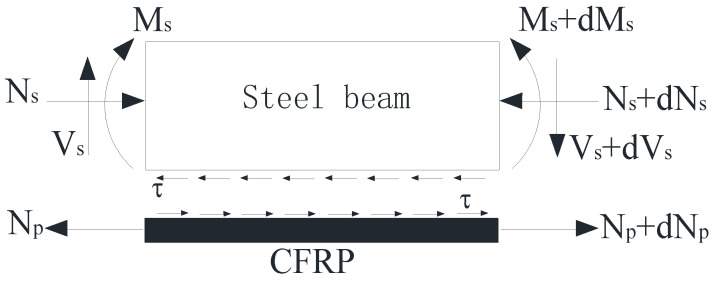
The force graph of unit body.

Based on the elastic method theory [34], according to the stress characteristics of steel beams strengthened with CFRP sheets, the following assumptions are made: (1) In normal use, the steel beam is an ideal elastomer. (2) The interface cemented layer only bears shear. (3) CFRP sheet only bears tension. (4) The section conforms to the assumption of plane section. The stress of steel beam element strengthened with CFRP sheet is shown in Figure 1., where *N_s_*, *V_s_*, and *M_s_* are the axial force, shear force, and bending moment of steel beam caused by external loading. *N_p_* is the axial force of CFRP sheet caused by external loading. τ is the interfacial shear stress.

The element equilibrium equation can be obtained from the force on the section.

(1) $$M_{s}-\left(M_{s}+dM_{s}\right)+V_{s}dx+\tau dx\cdot y_{s}=0$$

According to the relationship between bending moment and curvature

(2) $$\frac{d\phi }{dx}=\frac{1}{E_{s}I_{s}}\frac{dM_{s}}{dx}$$

Lower surface strain of lower flange of steel beam

(3) $$\varepsilon_{s}\left(x\right)=\phi y_{s}-\frac{N_{s}}{E_{s}A_{s}}=\phi y_{s}-\frac{N_{0}+P_{Ts}}{E_{s}A_{s}}$$

Upper surface strain of CFRP sheet

(4) $$\varepsilon_{p}\left(x\right)=\frac{N_{p}}{E_{p}\left(T,t\right)A_{p}}$$

where *N*_0_, *V_s_* and *M_s_* are the axial force, shear force and bending moment caused by external load, respectively. τ is the interfacial shear stress. $P_{Ts}$ is the axial force caused by temperature change [35]. $E_{p}\left(T,t\right)=\phi \left(T,t\right)E_{p}$. *E_p_(T,t)*, and $E_{p}$ are the elastic modulus and initial elastic modulus of CFRP sheet, respectively. ϕ(T,t) is the influence coefficient of temperature and creep [36].

So, the following can be available

(5) $$\frac{ds\left(x\right)}{dx}=\varepsilon_{s}\left(x\right)-\varepsilon_{p}\left(x\right)=y_{s}\phi -\left(\frac{N_{s}}{E_{s}A_{s}}+\frac{N_{p}}{E_{p}\left(T,t\right)A_{p}}\right)$$

### 2.2. Finite Element Verification

#### 2.2.1. Constitutive Relation

**Figure 2:**
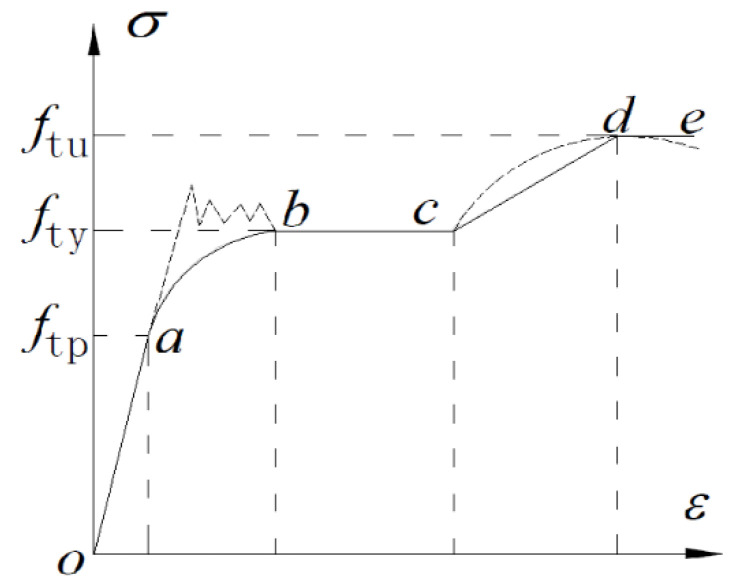
Stress-strain curve of steel.

**Figure 3:**
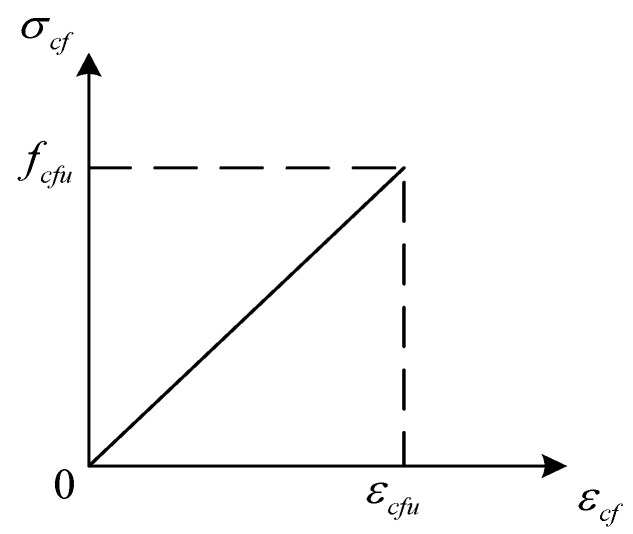
Stress-strain curve of CFRP sheet.

In order to verify the accuracy of the theoretical calculation, the finite element calculation software ABAQUS is used to check it. The constitutive relationship of steel beam in the ABAQUS model adopts the plastic analysis model. The constitutive relations of steel [37] and CFRP sheet [38] are given in Figure 2 and Figure 3. Poisson’s ratio of steel is taken as 0.3. CFRP sheet is an orthotropic material, and its strength is not taken into consideration in the direction perpendicular to the fiber. The stress-strain relationship curve of CFRP sheet is ideal elasticity. The elastic modulus of CFRP sheet is consistent with the value in the previous formula.

In Figure 2, $f_{tu}$ is the ultimate strength of steel. $f_{ty}$ is the yield strength of steel. $f_{tp}$ is the strength corresponding to proportional limit of steel. a is the proportional limit. b is the yield lower limit. c is the flow amplitude. d is the ultimate strength. e is the steel failure.

#### 2.2.2. Cell Selection and Mesh Generation

The CFRP cloth adopts the shell element (SR4) of four node reduced integral format, and adopts the Simpson integral of five integration points. The steel beam adopts the three-dimensional solid element (C3D8R) of eight node reduced integral format. The C3D8R element is more accurate in solving the displacement, and it reduces many degrees of freedom compared with the complete integration element, which can save calculation time.

**Figure 4:**
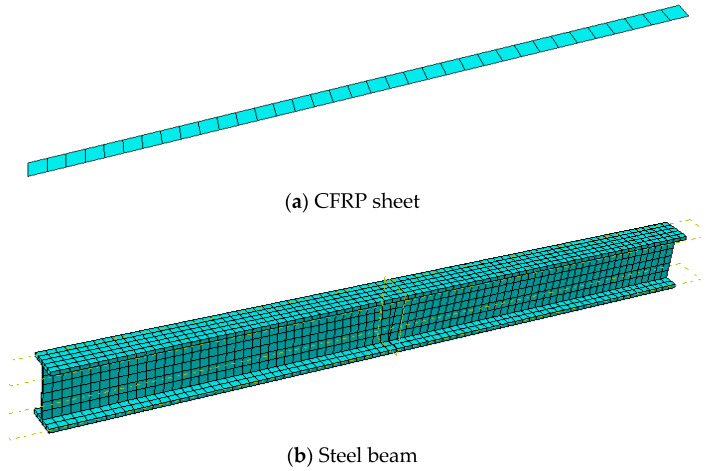
Element division.

The structural mesh generation method is used to discretize the components. Component mesh division is shown in Figure 4.

A steel cushion block with great rigidity shall be set at the load position and the support. Tie is used to constrain the loading end and the steel beam, and the steel beam and the support cushion block. In the calculation, the displacement loading is used to simulate the load action, and the incremental iteration method is used to solve the nonlinear equations.

#### 2.2.3. Calculation Results

**Figure 5:**
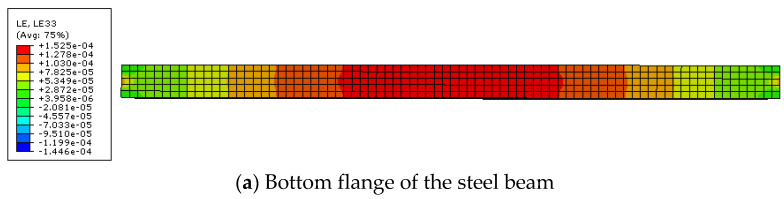

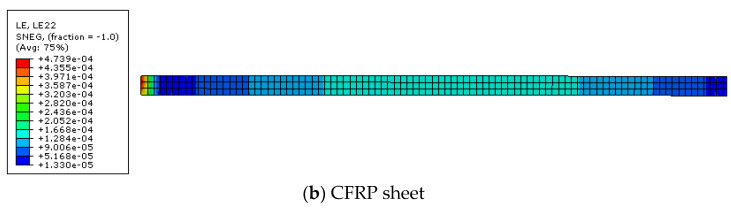
Strain distribution ata temperature of 20 °C.

**Figure 6:**
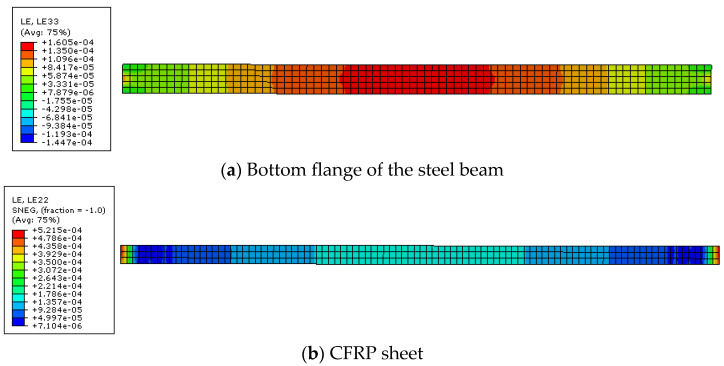
Strain distribution at a temperature of 25 °C.

Under the load of 10 N/mm and the temperature of 20 °C and 25 °C, the strain distribution of steel beam and CFRP sheet are shown in Figure 5 and Figure 6. Because the interface slip between steel beam and CFRP sheet cannot be obtained directly in ABAQUS, the difference of strain between them is used to calculate the interface slip between them.

**Figure 7:**
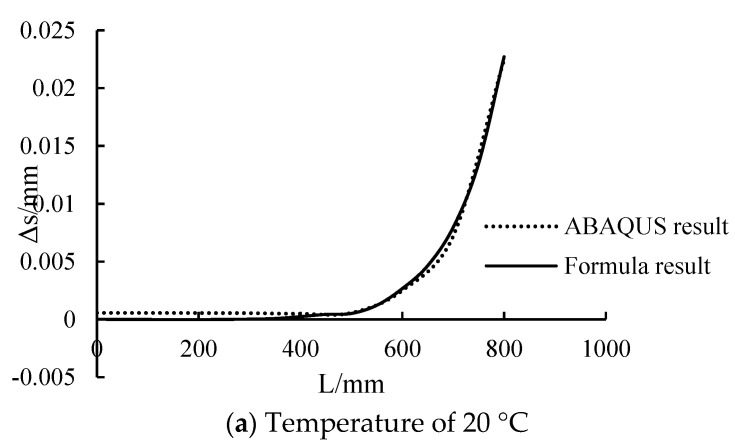

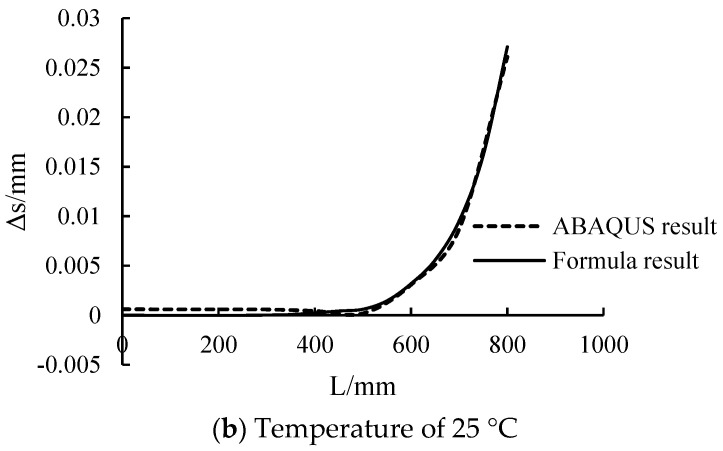
Comparison between numerical results and calculation results.

The comparison between finite element calculation and formula calculation results is shown in Figure 7. It can be seen from the figure that the two are in good agreement, indicating that the deduced formula is correct and can be used to calculate the interface slip between steel beam and CFRP sheet.

## 3. Tension Analysis of CFRP Sheet

### 3.1. Calculation of CFRP Sheet Tension

According to the relationship between CFRP cloth tension and interface slip [34]:

(6) $$\frac{ds\left(x\right)}{dx}=-\frac{1}{k_{a}}\frac{d^{2}N\left(x\right)}{dx^{2}}=y_{s}\phi -\left(\frac{N_{s}}{E_{s}A_{s}}+\frac{N_{p}}{E_{p}\left(T,t\right)A_{p}}\right)$$

$\phi =\frac{M_{s}}{E_{s}I_{s}}=\frac{M_{0}+M_{Ts}}{E_{s}I_{s}}$,$N_{s}=N_{p}$ available

(7) $$\frac{d^{2}N\left(x\right)}{dx^{2}}-k_{a}\left(\frac{1}{E_{s}A_{s}}+\frac{1}{E_{p}\left(T,t\right)A_{p}}\right)N\left(x\right)=-\frac{k_{a}y_{s}}{E_{s}A_{s}}\left(M_{0}+M_{Ts}\right)$$

(8) $$\frac{d^{2}N\left(x\right)}{dx^{2}}-\alpha_{N}^{2}N\left(x\right)=\beta_{N}\left(M_{0}+M_{Ts}\right)$$

where $\alpha_{N}^{2}=k_{a}\left(\frac{1}{E_{s}A_{s}}+\frac{1}{E_{p}\left(T,t\right)A_{p}}\right)$, $\beta_{N}=-\frac{k_{a}y_{s}}{E_{s}I_{s}}$. $M_{Ts}$ is the bending moment caused by temperature change [32]. $M_{Ts}=E_{s}\alpha_{s}\int_{h}t\left(y\right)b\left(y\right)\left(y-y_{s}\right)dy$.

#### 3.1.1. Uniform Load Action

According to the boundary conditions, the CFRP cloth tension under uniformly distributed load is

(9) $$N\left(x\right)=-\frac{\beta_{N}q+\alpha_{N}^{2}b_{p}k_{a}\left[\frac{P_{Ts}}{E_{s}A_{s}}-\frac{qy_{s}L\left(L-l_{f}\right)}{4E_{s}I_{s}}\right]}{\alpha_{N}^{4}\left(e^{\frac{\alpha_{N}l_{f}}{2}}+e^{-\frac{\alpha_{N}l_{f}}{2}}\right)}\left(e^{\alpha_{N}x}+e^{-\alpha_{N}x}\right)+\frac{\beta_{N}q}{2\alpha_{N}^{2}}x^{2}+\left[\frac{\left(8-\alpha_{N}^{2}L^{2}\right)\beta_{N}q}{8\alpha_{N}^{4}}+\frac{\beta_{N}M_{Ts}}{\alpha_{N}^{2}}\right]$$

#### 3.1.2. Symmetrical Concentrated Load Action

According to the boundary conditions, the tension of CFRP sheet under symmetrical concentrated load is:

Bending shear section

(10) $$\begin{array}{c} N_{1}\left(x\right)=\left\{-\frac{\beta_{N}Pe^{-\frac{\alpha_{N}l_{f}}{2}}}{4\alpha_{N}^{3}N_{1}}\left(e^{\frac{\alpha_{N}l_{0}}{2}}+e^{-\frac{\alpha_{N}l_{0}}{2}}\right)-\frac{b_{p}k_{a}\left[\frac{P_{Ts}}{E_{s}A_{s}}-\frac{Py_{s}\left(L-l_{f}\right)}{2E_{s}I_{s}}\right]e^{-\frac{\alpha_{N}l_{f}}{2}}}{2\alpha_{N}^{2}N_{1}}\left[N_{1}-e^{-\frac{\alpha_{N}\left(l_{f}-l_{0}\right)}{2}}\cdot e^{-\frac{\alpha_{N}l_{0}}{2}}\right]\right\}e^{\alpha_{N}x}\\ +\left\{\frac{\beta_{N}Pe^{\frac{\alpha_{N}l_{f}}{2}}}{4\alpha_{N}^{3}N_{1}}\left(e^{\frac{\alpha_{N}l_{0}}{2}}+e^{-\frac{\alpha_{N}l_{0}}{2}}\right)+\frac{b_{p}k_{a}e^{-\frac{\alpha_{N}l_{0}}{2}}\left[\frac{P_{Ts}}{E_{s}A_{s}}-\frac{Py_{s}\left(L-l_{f}\right)}{2E_{s}I_{s}}\right]e^{\frac{\alpha_{N}l_{0}}{2}}}{\alpha_{N}^{2}N_{1}}\right\}e^{-\alpha_{N}x}+\frac{\beta_{N}P}{\alpha_{N}^{2}}x-\left(\frac{\beta_{N}PL}{2\alpha_{N}^{2}}+\frac{\beta_{N}M_{Ts}}{\alpha_{N}^{2}}\right) \end{array}$$

Pure bending section

(11) $$N_{2}\left(x\right)=\left\{\frac{\beta_{N}P\left[e^{\frac{\alpha_{N}\left(l_{f}-l_{0}\right)}{2}}-e^{-\frac{\alpha_{N}\left(l_{f}-l_{0}\right)}{2}}\right]}{2\alpha_{N}^{3}\left(e^{\frac{\alpha_{N}l_{f}}{2}}+e^{-\frac{\alpha_{N}l_{f}}{2}}\right)}-\frac{b_{p}k_{a}\left[\frac{P_{Ts}}{E_{s}A_{s}}-\frac{Py_{s}\left(L-l_{f}\right)}{2E_{s}I_{s}}\right]}{2\alpha_{N}^{2}\left(e^{\frac{\alpha_{N}l_{f}}{2}}+e^{-\frac{\alpha_{N}l_{f}}{2}}\right)}\right\}\left(e^{\alpha_{N}x}+e^{-\alpha_{N}x}\right)-\left[\frac{\beta_{N}P\left(L-l_{0}\right)}{2\alpha_{N}^{2}}+\frac{\beta_{N}M_{Ts}}{\alpha_{N}^{2}}\right]$$

where

$$N_{1}=\frac{1}{4}\left[e^{\frac{\alpha_{N1}\left(l_{f}-l_{0}\right)}{2}}-e^{-\frac{\alpha_{N1}\left(l_{f}-l_{0}\right)}{2}}\right]\left(e^{\frac{\alpha_{N1}l_{0}}{2}}-e^{-\frac{\alpha_{N1}l_{0}}{2}}\right)+\frac{1}{4}\left[e^{\frac{\alpha_{N1}\left(l_{f}-l_{0}\right)}{2}}+e^{-\frac{\alpha_{N1}\left(l_{f}-l_{0}\right)}{2}}\right]\left(e^{\frac{\alpha_{N1}l_{0}}{2}}+e^{-\frac{\alpha_{N1}l_{0}}{2}}\right)$$

#### 3.1.3. Arbitrary Concentrated Load Action

According to the boundary conditions, the CFRP sheet tension under any concentrated load is:

Left side of loading point

(12) $$N_{1}\left(x\right)=-\frac{\beta_{N}P\left(L-2b\right)e^{-\frac{\alpha_{N}l_{f}}{2}}+2\alpha_{N}b_{f}k_{a}\left[\frac{P_{Ts}}{E_{s}A_{s}}-\frac{y_{s}\left(L-l_{f}\right)}{2E_{s}I_{s}}\left(\frac{P}{2}-\frac{Pb}{L}\right)\right]}{2L\alpha_{N}^{3}\left(e^{\frac{\alpha_{N}l_{f}}{2}}+e^{-\frac{\alpha_{N}l_{f}}{2}}\right)}e^{\alpha_{N}x}-\frac{\beta_{N}P\left(L-2b\right)}{2L\alpha_{N}^{2}}x-\left(\frac{\beta_{N}P\left(L-2b\right)}{4\alpha_{N}^{2}}+\beta_{N}M_{Ts}\right)\;+\frac{\beta_{N}P\left(L-2b\right)e^{\frac{\alpha_{N}l_{f}}{2}}-2\alpha_{N}b_{f}k_{a}\left[\frac{P_{Ts}}{E_{s}A_{s}}-\frac{y_{s}\left(L-l_{f}\right)}{2E_{s}I_{s}}\left(\frac{P}{2}-\frac{Pb}{L}\right)\right]}{2L\alpha_{N}^{3}\left(e^{\frac{\alpha_{N}l_{f}}{2}}+e^{-\frac{\alpha_{N}l_{f}}{2}}\right)}e^{-\alpha_{N}x}$$

Right side of loading point

(13) $$N_{2}\left(x\right)=-\frac{\beta_{N}P\left(L+2b\right)e^{-\frac{\alpha_{N}l_{f}}{2}}+2\alpha_{N}b_{f}k_{a}\left[\frac{P_{Ts}}{E_{s}A_{s}}-\frac{y_{s}\left(L-l_{f}\right)}{2E_{s}I_{s}}\left(\frac{P}{2}+\frac{Pb}{L}\right)\right]}{2L\alpha_{N}^{3}\left(e^{\frac{\alpha_{N}l_{f}}{2}}+e^{-\frac{\alpha_{N}l_{f}}{2}}\right)}e^{\alpha_{N}x}-\frac{\beta_{N}P\left(L+2b\right)}{2L\alpha_{N}^{2}}x-\left(\frac{\beta_{N}P\left(L+2b\right)}{4\alpha_{N}^{2}}+\beta_{N}M_{Ts}\right)\;+\frac{\beta_{N}P\left(L+2b\right)e^{\frac{\alpha_{N}l_{f}}{2}}-2\alpha_{N}b_{f}k_{a}\left[\frac{P_{Ts}}{E_{s}A_{s}}-\frac{y_{s}\left(L-l_{f}\right)}{2E_{s}I_{s}}\left(\frac{P}{2}+\frac{Pb}{L}\right)\right]}{2L\alpha_{N}^{3}\left(e^{\frac{\alpha_{N}l_{f}}{2}}+e^{-\frac{\alpha_{N}l_{f}}{2}}\right)}e^{-\alpha_{N}x}$$

### 3.2. Design Parameter Analysis

#### 3.2.1. Adhesive Layer Stiffness

**Figure 8:**
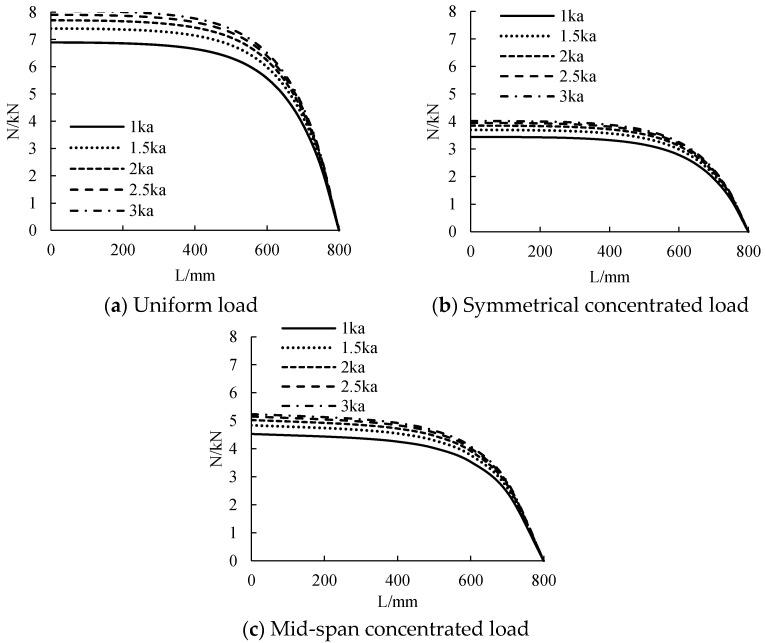
The influence of adhesive layer stiffness on tensile force.

The distribution curve of CFRP sheet tension along the beam length under different stiffness is calculated, as shown in Figure 8. As can be seen from the figure, the tensile force of CFRP sheet is nonlinear along the beam length. The tensile force of mid-span CFRP sheet is the largest, and the beam end tends to 0. When the stiffness changes from 1 ka to 3 ka, the tensile force of CFRP sheet increases with the increase of connection stiffness. Under uniform load, the CFRP sheet tension at stiffness of 1.5 ka, 2 ka, 2.5 ka and 3 ka increases by 7.39%, 10.30%, 14.66% and 16.40%, respectively, compared with 1 ka. Under symmetrical concentrated load, the CFRP sheet tension at stiffness of 1.5 ka, 2 ka, 2.5 ka and 3 ka increases by 7.53%, 11.92%, 14.83% and 16.86%, respectively, compared with 1 ka. Under mid-span concentrated load, the CFRP sheet tension at stiffness of 1.5 ka, 2 ka, 2.5 ka and 3 ka increases by 6.84%, 10.82%, 13.47% and 15.45%, respectively, compared with 1 ka. The greater the increase of connection stiffness, the smaller the increase of CFRP tensile force. When the stiffness reaches about 3 ka, the stiffness has little influence on the tensile change of the CFRP sheet.

#### 3.2.2. Temperature

**Figure 9:**
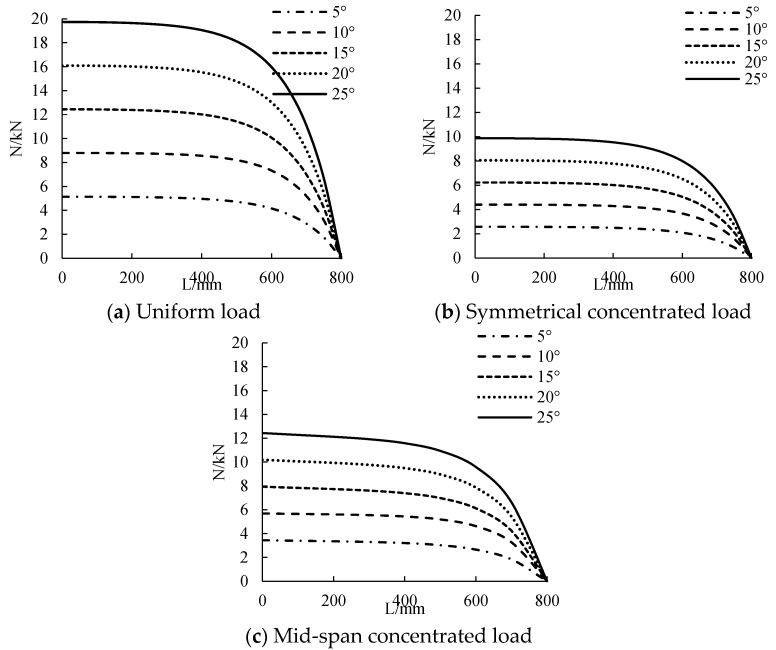
The influence of temperature on tensile force.

The distribution curve of CFRP sheet tension along the beam length at different temperatures is calculated, as shown in Figure 9. The distribution of CFRP tensile force along the beam length is nonlinear, and the maximum is in the middle of the span and the minimum at the beam end tends to 0. When the temperature is from 0 °C to 25 °C, the tensile force of CFRP sheet increases with the increase of temperature. The higher the temperature, the steeper is the distribution of the tensile curve of CFRP sheet. When the temperature is low, the distribution of the CFRP sheet tension curve is relatively flat. As the temperature is increased from 5 °C to 25 °C, under the action of uniform load, symmetrical concentrated load and mid-span concentrated load, the CFRP sheet tension increases by about 3.7 kN, 1.8 kN and 2.3 kN, respectively. This shows that temperature has a great influence on the tension of CFRP sheet.

#### 3.2.3. CFRP Sheet Thickness

**Figure 10:**
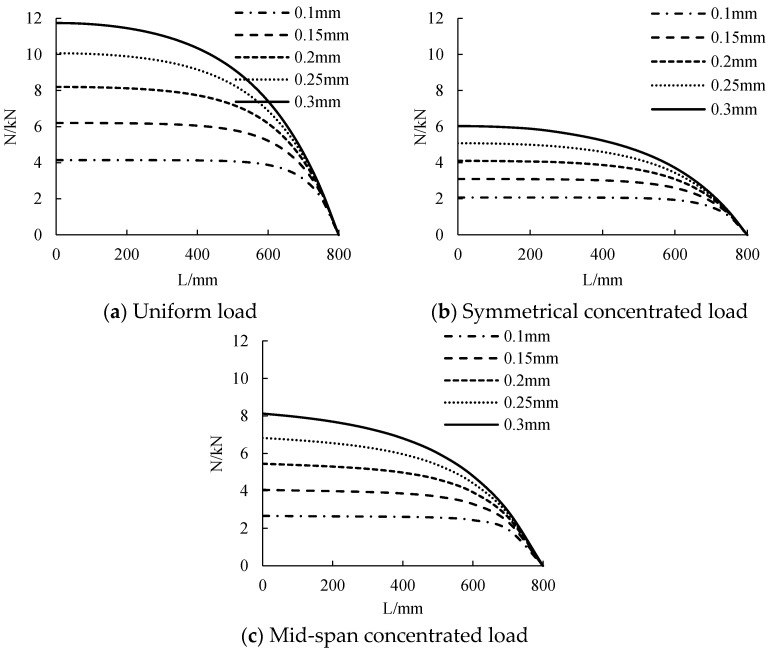
The influence of CFRP sheet’s thickness on tensile force.

The distribution curve of CFRP cloth tension along the beam length under different CFRP cloth thickness is calculated, as shown in Figure 10. The distribution of CFRP tensile force along the beam length is nonlinear, and the maximum is in the middle of the span and the minimum at the beam end tends to 0. When the thickness of CFRP sheet is from 0.1 mm to 0.3 mm, the tensile strength of CFRP sheet increases with the increase of the thickness of CFRP sheet. The greater the thickness of CFRP sheet, the steeper is the distribution of the CFRP sheet tension curve. When the thickness of CFRP sheet is small, the distribution of the tension curve of CFRP sheet is relatively flat. When the thickness of CFRP sheet changes from 0.1 mm to 0.3 mm, the tension of CFRP sheet will increase by about 1.9 kN, 1 kN and 1.4 kN for each 0.05 mm increase in the width of CFRP sheet under uniform load, symmetrical concentrated load and mid-span concentrated load.

#### 3.2.4. Load under Creep

**Figure 11:**
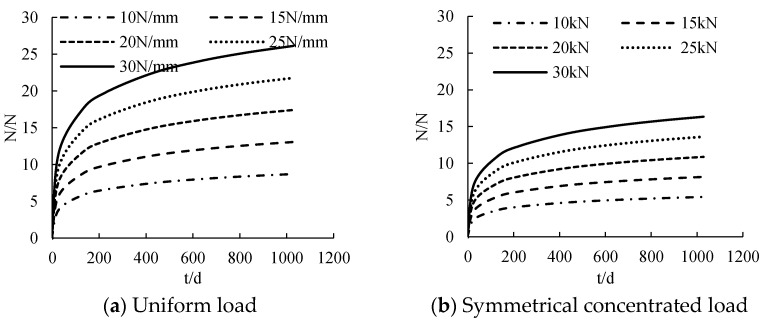

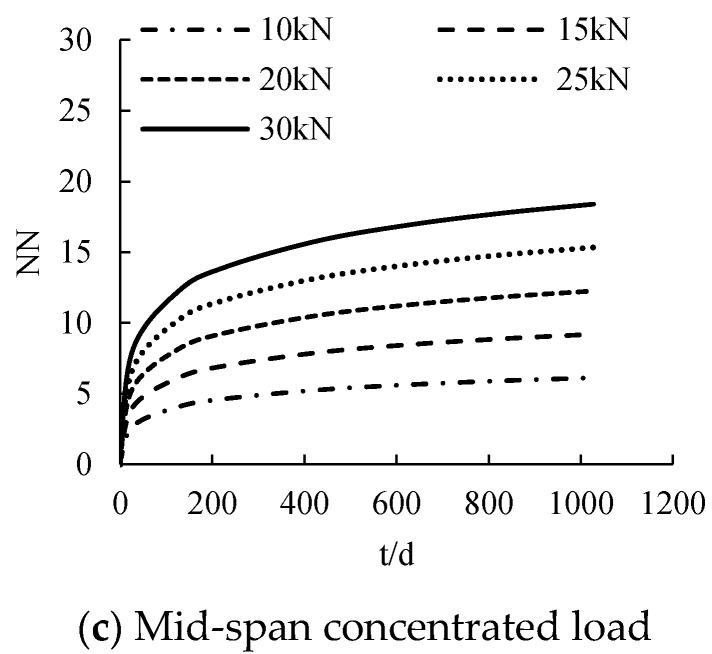
The influence of loading under creep on tensile force.

The distribution curve of CFRP sheet tension with time under different loads is calculated, as shown in Figure 11. CFRP sheet tension has a nonlinear distribution with the increase of time. The CFRP sheet tension at the initial stage of loading is 0. With the increase of age, the tensile force of CFRP increases gradually. The tension of CFRP sheet increases according to the increase of load. The greater the load is, the steeper the slip curve is. When the load changes from 10 N/mm to 30 N/mm, the CFRP sheet tension increases by about 4.4 N, 2.7 N and 3.1 N, respectively, for each 5 kN increase in load under the action of uniform load, symmetrical concentrated load and mid-span concentrated load.

#### 3.2.5. Stiffness of Adhesive Layer under Creep

**Figure 12:**
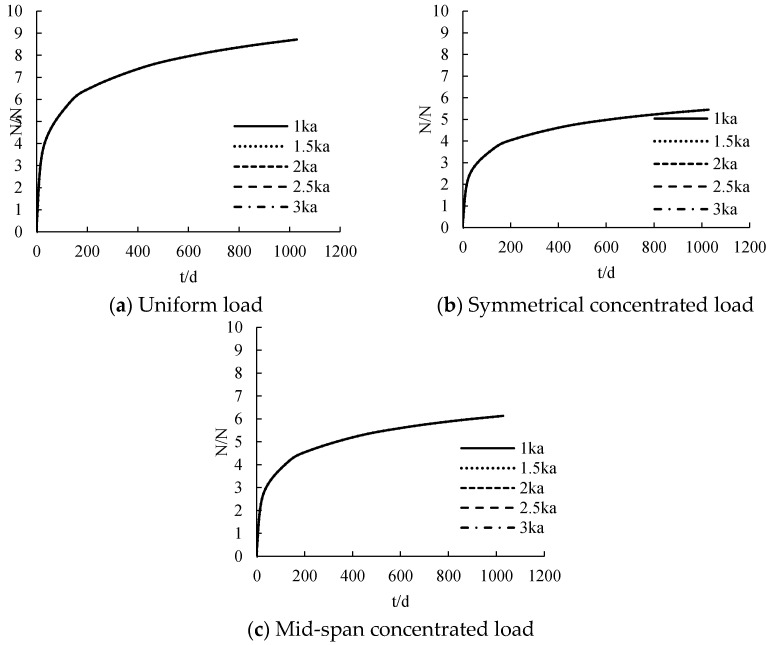
The influence of adhesive layer stiffness under creep on tensile force.

The distribution curve of CFRP sheet tension with time under different stiffness is calculated, as shown in Figure 12. Under uniform load, symmetrical concentrated load and mid-span concentrated load, the CFRP sheet tension is nonlinear with the increase of time. The CFRP sheet tension increases rapidly within 28 days, and the growth rate becomes relatively slow after 28 days. After about 300 d, the CFRP sheet tension tends to be stable. CFRP sheet tension increases according to the increase of connection stiffness. However, the change is absolutely subtle. The greater the increase of stiffness, the smaller is the increase of CFRP sheet tension.

## 4. Deformation Analysis of Steel Beam

### 4.1. Deformation Calculation of Steel Beam

According to the relationship between bending moment and curvature $\frac{M}{EI}=-\frac{d^{2}W\left(x\right)}{dx^{2}}$, and combined with the previous results, the calculation formula of deformation can be obtained.

#### 4.1.1. Uniform Load Action

According to the boundary conditions, the deformation under uniform load is

(14) $$W\left(x\right)=\frac{C_{1}y_{s}}{\alpha_{1}^{3}E_{s}I_{s}}\left(e^{\alpha_{1}x}+e^{-\alpha_{1}x}\right)+\frac{\left(\alpha_{1}^{2}-\beta_{1}y_{s}\right)q}{24\alpha_{1}^{2}E_{s}I_{s}}x^{4}+\frac{1}{2}\left(\frac{2y_{s}}{\alpha_{1}E_{s}I_{s}}C_{1}-\frac{qL^{2}}{8E_{s}I_{s}}\right)x^{2}+\frac{qL^{4}}{384E_{s}I_{s}}-\frac{L^{2}}{8}\left(\frac{2y_{s}}{\alpha_{1}E_{s}I_{s}}C_{1}-\frac{qL^{2}}{8E_{s}I_{s}}\right)$$

where $C_{1}=\frac{\frac{P_{Ts}}{E_{s}A_{s}}-\frac{qy_{s}L\left(L-l_{f}\right)}{8E_{s}I_{s}}-\frac{y_{s}q}{\alpha_{1}^{2}E_{s}I_{s}}}{\alpha_{1}\left(e^{\frac{\alpha_{1}l_{f}}{2}}+e^{-\frac{\alpha_{1}l_{f}}{2}}\right)}$,$\alpha_{1}^{2}=k_{a}\left(\frac{1}{E_{s}A_{s}}+\frac{y_{s}^{2}}{E_{s}I_{s}}+\frac{1}{E_{p}\left(T,t\right)A_{p}}\right)$,$\beta_{1}=\frac{k_{a}y_{s}}{E_{s}I_{s}}$.

#### 4.1.2. Symmetrical Concentrated Load Action

According to the boundary conditions, the deformation under symmetrical concentrated load is:

Bending shear section

(15) $$\begin{array}{c} W_{1}\left(x\right)=\frac{y_{s}}{\alpha_{1}^{3}E_{s}I_{s}}(C_{1}e^{\alpha_{1}x}-C_{2}e^{-\alpha_{1}x})-\frac{\beta_{1}Py_{s}}{6\alpha_{1}^{2}E_{s}I_{s}}x^{3}+\frac{P}{6E_{s}I_{s}}x^{3}+\frac{x^{2}}{2}\left\{\frac{y_{s}\left[\frac{P_{Ts}}{E_{s}A_{s}}-\frac{Py_{s}\left(L-l_{f}\right)}{2E_{s}I_{s}}\right]}{\alpha_{1}^{2}E_{s}I_{s}}-\frac{\beta_{1}Py_{s}l_{f}}{2\alpha_{1}^{2}E_{s}I_{s}}-\frac{PL}{2E_{s}I_{s}}\right\}+\left(x-\frac{L}{2}\right)\\ \left\{\frac{-y_{s}}{\alpha_{1}^{2}E_{s}I_{s}}[\left(C_{1}-C_{6}\right)e^{\frac{\alpha_{1}l_{0}}{2}}+\left(C_{2}-C_{7}\right)e^{-\frac{\alpha_{1}l_{0}}{2}}]+\frac{\left(\beta_{1}y_{s}-\alpha_{1}^{2}\right)Pl_{0}^{2}}{8\alpha_{1}^{2}E_{s}I_{s}}+\frac{l_{0}}{2}\left(-\frac{2y_{s}}{\alpha_{1}E_{s}I_{s}}C_{6}-\frac{P\left(L-l_{0}\right)}{2E_{s}I_{s}}-\frac{y_{s}\left[\frac{P_{Ts}}{E_{s}A_{s}}-\frac{Py_{s}\left(L-l_{f}\right)}{2E_{s}I_{s}}\right]}{\alpha_{1}^{2}E_{s}I_{s}}+\frac{\beta_{1}Py_{s}l_{f}}{2\alpha_{1}^{2}E_{s}I_{s}}+\frac{PL}{2E_{s}I_{s}}\right)\right\}\\ -\frac{PL^{3}}{48E_{s}I_{s}}-\frac{L^{2}}{8}\left\{\frac{y_{s}\left[\frac{P_{Ts}}{E_{s}A_{s}}-\frac{Py_{s}\left(L-l_{f}\right)}{2E_{s}I_{s}}\right]}{\alpha_{1}^{2}E_{s}I_{s}}-\frac{\beta_{1}Py_{s}l_{f}}{2\alpha_{1}^{2}E_{s}I_{s}}-\frac{PL}{2E_{s}I_{s}}\right\} \end{array}$$

Pure bending section

(16) $$\begin{array}{c} W_{2}\left(x\right)=\frac{y_{s}}{\alpha_{1}^{3}E_{s}I_{s}}\left(C_{6}e^{\alpha_{1}x}-C_{7}e^{-\alpha_{1}x}\right)-\frac{x^{2}}{2}\left[\frac{2y_{s}}{\alpha_{1}E_{s}I_{s}}C_{6}+\frac{P\left(L-l_{0}\right)}{2E_{s}I_{s}}\right]+\frac{y_{s}}{\alpha_{1}^{3}E_{s}I_{s}}\left(C_{1}e^{\frac{\alpha_{1}l_{0}}{2}}-C_{2}e^{-\frac{\alpha_{1}l_{0}}{2}}\right)-\frac{y_{s}\beta_{1}Pl_{0}^{3}}{48\alpha_{1}^{2}E_{s}I_{s}}+\frac{P\left(l_{0}^{3}-L^{3}\right)}{48E_{s}I_{s}}\\ +\frac{1}{8}\left(l_{0}^{2}-L^{2}\right)\left\{\frac{y_{s}\left[\frac{P_{Ts}}{E_{s}A_{s}}-\frac{Py_{s}\left(L-l_{f}\right)}{2E_{s}I_{s}}\right]}{\alpha_{1}^{2}E_{s}I_{s}}-\frac{\beta_{1}Py_{s}l_{f}}{2\alpha_{1}^{2}E_{s}I_{s}}-\frac{PL}{2E_{s}I_{s}}\right\}-\frac{y_{s}}{\alpha_{1}^{3}E_{s}I_{s}}\left(C_{6}e^{\frac{\alpha_{1}l_{0}}{2}}-C_{7}e^{-\frac{\alpha_{1}l_{0}}{2}}\right)+\frac{l_{0}^{2}}{8}\left[\frac{2y_{s}}{\alpha_{1}E_{s}I_{s}}C_{6}+\frac{P\left(L-l_{0}\right)}{2E_{s}I_{s}}\right]\\ +\frac{l_{0}-L}{2}\left\{\frac{-y_{s}}{\alpha_{1}^{2}E_{s}I_{s}}\left[\left(C_{1}-C_{6}\right)e^{\frac{\alpha_{1}l_{0}}{2}}+\left(C_{2}-C_{7}\right)e^{-\frac{\alpha_{1}l_{0}}{2}}\right]+\frac{\left(\beta_{1}y_{s}-\alpha_{1}^{2}\right)Pl_{0}^{2}}{8\alpha_{1}^{2}E_{s}I_{s}}+\frac{l_{0}}{2}\left(-\frac{2y_{s}}{\alpha_{1}E_{s}I_{s}}C_{6}-\frac{P\left(L-l_{0}\right)}{2E_{s}I_{s}}-\frac{y_{s}\left[\frac{P_{Ts}}{E_{s}A_{s}}-\frac{Py_{s}\left(L-l_{f}\right)}{2E_{s}I_{s}}\right]}{\alpha_{1}^{2}E_{s}I_{s}}+\frac{\beta_{1}Py_{s}l_{f}}{2\alpha_{1}^{2}E_{s}I_{s}}+\frac{PL}{2E_{s}I_{s}}\right)\right\} \end{array}$$

where $C_{1}=\frac{\beta_{1}P\left[e^{\frac{\alpha_{1}}{2}\left(l_{0}-l_{f}\right)}+e^{-\frac{\alpha_{1}}{2}\left(l_{0}+l_{f}\right)}\right]+2\alpha_{1}\left[\frac{P_{Ts}}{E_{s}A_{s}}-\frac{Py_{s}\left(L-l_{f}\right)}{2E_{s}I_{s}}\right]}{2\alpha_{1}^{2}\left(e^{\frac{\alpha_{1}l_{f}}{2}}+e^{-\frac{\alpha_{1}l_{f}}{2}}\right)}$, $C_{2}=\frac{\beta_{1}P\left[e^{\frac{\alpha_{1}}{2}\left(l_{0}+l_{f}\right)}+e^{-\frac{\alpha_{1}}{2}\left(l_{0}-l_{f}\right)}\right]-2\alpha_{1}\left[\frac{P_{Ts}}{E_{s}A_{s}}-\frac{Py_{s}\left(L-l_{f}\right)}{2E_{s}I_{s}}\right]}{2\alpha_{1}^{2}\left(e^{\frac{\alpha_{1}l_{f}}{2}}+e^{-\frac{\alpha_{1}l_{f}}{2}}\right)}$, $C_{6}=-C_{7}=\frac{2\alpha_{1}\left[\frac{P_{Ts}}{E_{s}A_{s}}-\frac{Py_{s}\left(L-l_{f}\right)}{2E_{s}I_{s}}\right]-\beta_{1}P\left[e^{\alpha_{1}\left(\frac{l_{f}}{2}-\frac{l_{0}}{2}\right)}-e^{-\alpha_{1}\left(\frac{l_{f}}{2}-\frac{l_{0}}{2}\right)}\right]}{2\alpha_{1}^{2}\left(e^{\frac{\alpha_{1}l_{f}}{2}}+e^{-\frac{\alpha_{1}l_{f}}{2}}\right)}$.

#### 4.1.3. Arbitrary Concentrated Load Action

According to the boundary conditions, the deformation under any concentrated load can be obtained as:

Left side of loading point

(17) $$\begin{array}{cc} W_{1} & \left(x\right)=\frac{y_{s}}{\alpha_{1}^{3}E_{s}I_{s}}\left(C_{1}e^{\alpha_{1}x}-C_{2}e^{-\alpha_{1}x}\right)-\frac{\beta_{1}y_{s}P\left(L-2b\right)}{12L\alpha_{1}^{2}E_{s}I_{s}}x^{3}+\frac{P\left(L-2b\right)}{12LE_{s}I_{s}}x^{3}-\frac{1}{2}\left[\frac{y_{s}}{\alpha_{1}^{}E_{s}I_{s}}\left(C_{1}-C_{2}\right)+\frac{L}{2E_{s}I_{s}}\left(\frac{P}{2}-\frac{Pb}{L}\right)\right]x^{2}\\ & -\left\{\frac{y_{s}\beta_{1}}{\alpha_{1}^{4}E_{s}I_{s}}\left(\frac{P}{2}-\frac{Pb}{L}\right)\right\}x-\frac{L^{3}}{48E_{s}I_{s}}\left(\frac{P}{2}-\frac{Pb}{L}\right)+\frac{L^{2}}{8}\left[\frac{y_{s}}{\alpha_{1}^{}E_{s}I_{s}}\left(C_{1}-C_{2}\right)+\frac{L}{2E_{s}I_{s}}\left(\frac{P}{2}-\frac{Pb}{L}\right)\right]+\frac{L}{2}\left[\frac{y_{s}\beta_{1}}{\alpha_{1}^{4}E_{s}I_{s}}\left(\frac{P}{2}-\frac{Pb}{L}\right)\right] \end{array}$$

Right side of loading point

(18) $$\begin{array}{cc} W_{2} & \left(x\right)=\frac{y_{s}}{\alpha_{1}^{3}E_{s}I_{s}}\left(C_{6}e^{\alpha_{1}x}-C_{7}e^{-\alpha_{1}x}\right)-\frac{\beta_{1}y_{s}P\left(L+2b\right)}{12L\alpha_{1}^{2}E_{s}I_{s}}x^{3}+\frac{P\left(L+2b\right)}{12LE_{s}I_{s}}x^{3}-\frac{1}{2}\left[\frac{y_{s}}{\alpha_{1}^{}E_{s}I_{s}}\left(C_{6}-C_{7}\right)+\frac{L}{2E_{s}I_{s}}\left(\frac{P}{2}+\frac{Pb}{L}\right)\right]x^{2}\\ & -\left[\frac{y_{s}\beta_{1}}{\alpha_{1}^{4}E_{s}I_{s}}\left(\frac{P}{2}+\frac{Pb}{L}\right)\right]x-\frac{L^{3}}{48E_{s}I_{s}}\left(\frac{P}{2}+\frac{Pb}{L}\right)+\frac{L^{2}}{8}\left[\frac{y_{s}}{\alpha_{1}^{}E_{s}I_{s}}\left(C_{6}-C_{7}\right)+\frac{L}{2E_{s}I_{s}}\left(\frac{P}{2}+\frac{Pb}{L}\right)\right]+\frac{L}{2}\left[\frac{y_{s}\beta_{1}}{\alpha_{1}^{4}E_{s}I_{s}}\left(\frac{P}{2}+\frac{Pb}{L}\right)\right] \end{array}$$

where $C_{1}=\frac{\frac{P_{Ts}}{E_{s}A_{s}}-\frac{y_{s}\left(L-l_{f}\right)}{2E_{s}I_{s}}\left(\frac{P}{2}-\frac{Pb}{L}\right)+\frac{\beta_{1}e^{-\frac{\alpha_{1}l_{f}}{2}}}{\alpha_{1}}\left(\frac{P}{2}-\frac{Pb}{L}\right)}{\alpha_{1}\left(e^{\frac{\alpha_{1}l_{f}}{2}}+e^{-\frac{\alpha_{1}l_{f}}{2}}\right)}$, $C_{2}=-C_{1}+\frac{\beta_{1}}{\alpha_{1}^{2}}\left(\frac{P}{2}-\frac{Pb}{L}\right)$, $C_{6}=\frac{\frac{P_{Ts}}{E_{s}A_{s}}-\frac{y_{s}\left(L-l_{f}\right)}{2E_{s}I_{s}}\left(\frac{P}{2}+\frac{Pb}{L}\right)+\frac{\beta_{1}e^{-\frac{\alpha_{1}l_{f}}{2}}}{\alpha_{1}}\left(\frac{P}{2}+\frac{Pb}{L}\right)}{\alpha_{1}\left(e^{\frac{\alpha_{1}l_{f}}{2}}+e^{-\frac{\alpha_{1}l_{f}}{2}}\right)}$, $C_{7}=-C_{6}+\frac{\beta_{1}}{\alpha_{1}^{2}}\left(\frac{P}{2}+\frac{Pb}{L}\right)$.

### 4.2. Design Parameter Analysis

#### 4.2.1. Adhesive Layer Stiffness

**Figure 13:**
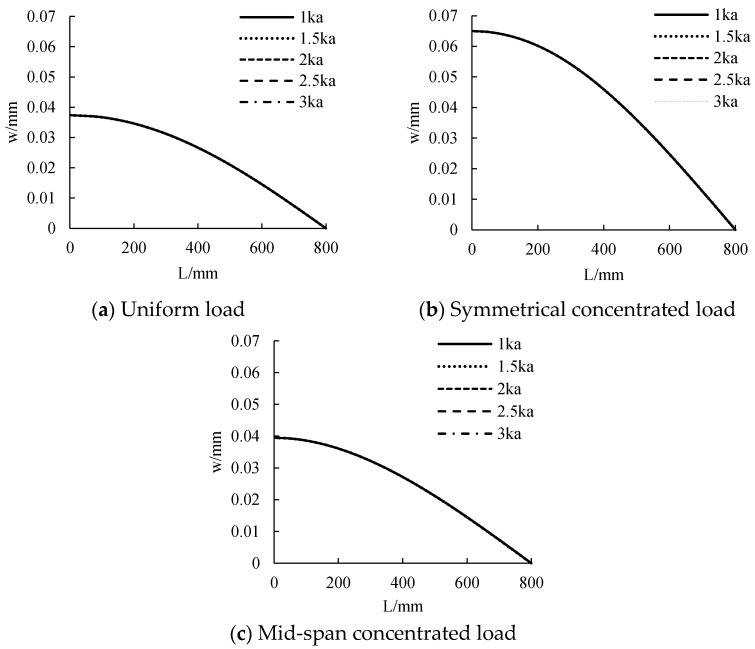
The influence of adhesive layer stiffness on displacement.

The distribution curve of deformation along the beam length under different stiffness is calculated, as shown in Figure 13. As can be seen from the figure, the deformation presents a nonlinear distribution along the beam length. The deformation decreases with the increase of stiffness, but the reduction range is very small. It shows that the effect of stiffness on reducing member deformation and improving its bearing capacity is not obvious. Under uniform load, symmetrical concentrated load and mid-span concentrated load, the deformation is 0.037 mm, 0.065 mm and 0.039 mm, respectively.

#### 4.2.2. Temperature

**Figure 14:**
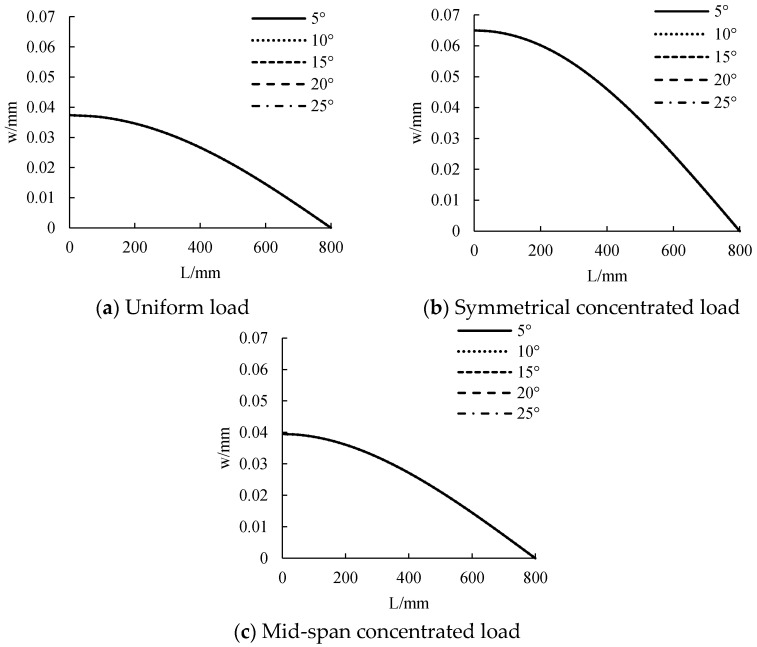
The influence of temperature on displacement.

The distribution curve of deformation along the beam length under different temperatures is calculated, as shown in Figure 14. The deformation increases with the increase of temperature from 0 °C to 25 °C. The increase is not very large, however. Thus, the influence of temperature on member deformation and bearing capacity is not obvious. Under uniform load, symmetrical concentrated load and mid-span concentrated load, the deformation is 0.037 mm, 0.065 mm and 0.039 mm, respectively.

#### 4.2.3. CFRP Sheet Thickness

**Figure 15:**
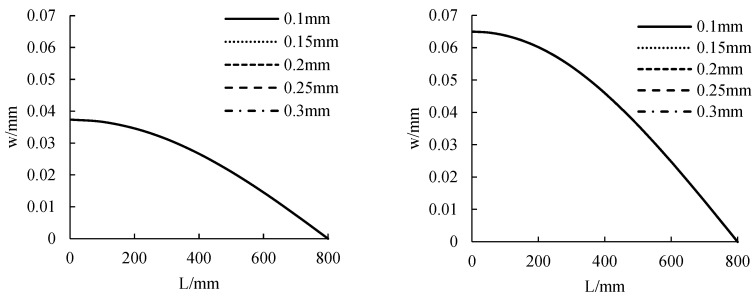

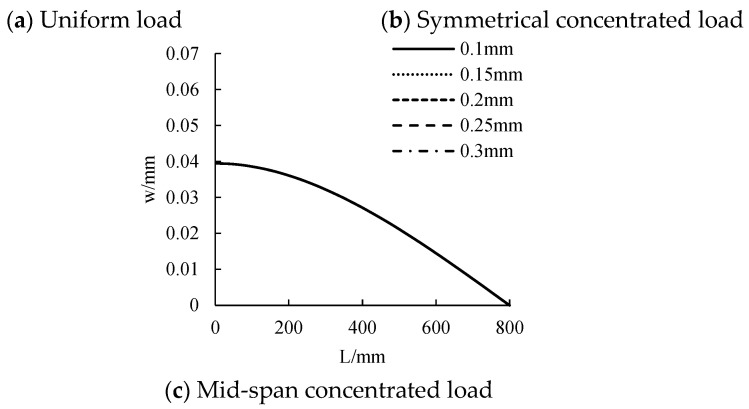
The influence of CFRP sheet’s thickness on displacement.

The deformation distribution curve along the beam length under different CFRP sheet thickness is calculated, as shown in Figure 15. When the thickness of CFRP sheet is from 0.1 mm to 0.3 mm, the deformation decreases with the increase of the thickness of CFRP sheet. The reduction is not obvious, however. It shows that the change of CFRP sheet thickness has no obvious effect on the deformation and bearing capacity of members. Under uniform load, symmetrical concentrated load and mid-span concentrated load, the deformation is 0.037 mm, 0.065 mm and 0.039 mm, respectively.

#### 4.2.4. Load under Creep

**Figure 16:**
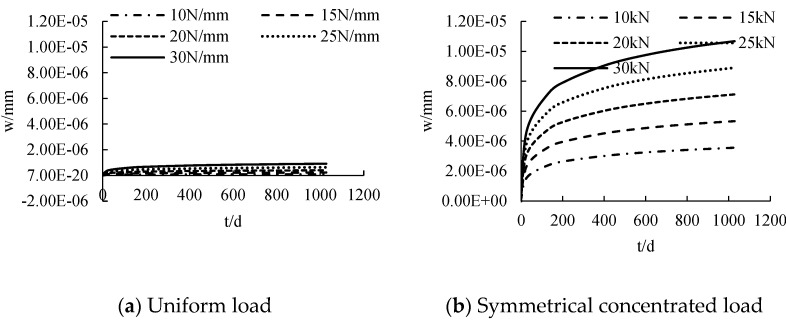

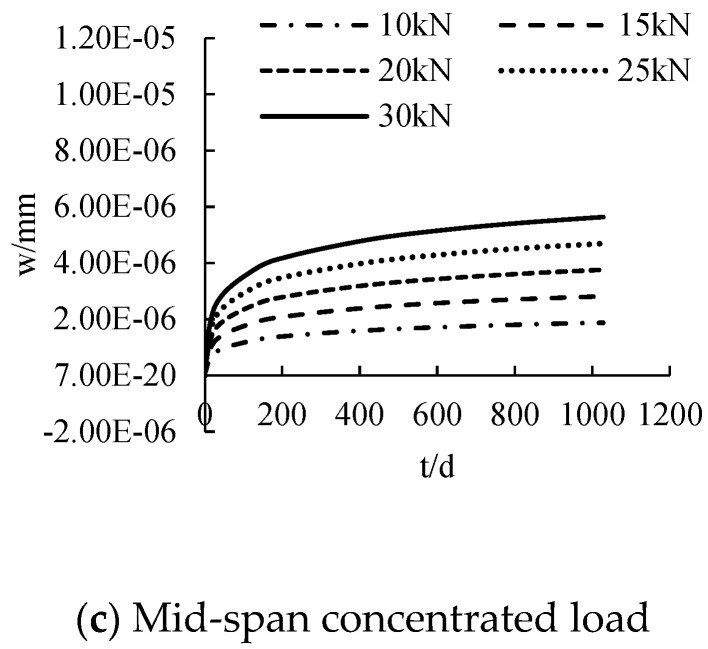
The influence of loading under creep on displacement.

The distribution curve of deformation with time under different loads is calculated, as shown in Figure 16. The deformation is nonlinear. The deformation at the initial stage of loading is 0. With the increase of age, the deformation increases gradually. The deformation increases with the increase of load. The greater the load, the steeper is the slip curve. When the load increases by 5 kN, the deformation increases by about 2.2 × 10^−7^ mm, 1.8 × 10^−6^ mm, 9.4 × 10^−7^ mm, respectively. Under the uniform load, the deformation of 15 N/mm, 20 N/mm, 25 N/mm and 30 N/mm load increases 125.17%, 302.08%, 503.74% and 799.71%, respectively, compared with the deformation of 10 N/mm load. Under the symmetrical concentrated load, the deformation of 15 N/mm, 20 N/mm, 25 N/mm and 30 N/mm load increases 50.02%, 100.05%, 141.29% and 182.87%, respectively, compared with the deformation of 10 N/mm load. Under the mid-span concentrated load, the deformation of 15 N/mm, 20 N/mm, 25 N/mm and 30 N/mm load increases 50.28%, 101.11%, 159.67% and 200.55%, respectively, compared with the deformation of 10 N/mm load.

#### 4.2.5. Stiffness of Adhesive Layer under Creep

**Figure 17:**
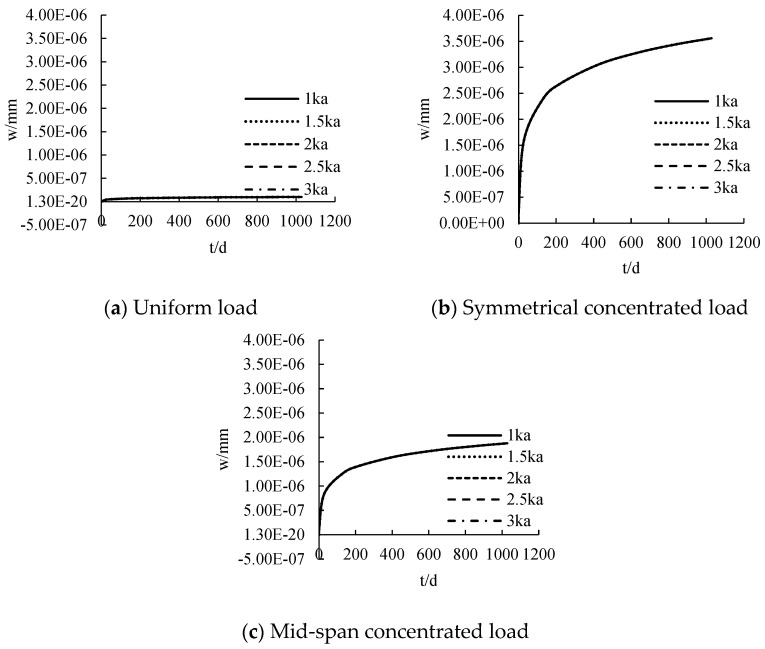
The influence of adhesive layer stiffness under creep on displacement.

The distribution curve of deformation with time under different stiffness is calculated, as shown in Figure 17. Under the action of uniform load, symmetrical concentrated load and mid-span concentrated load, the deformation presents a nonlinear distribution with the increase of time. The deformation increases rapidly within 28 days, and the growth rate becomes relatively slow after 28 days. The deformation tends to be stable after about 300 d. Under the action of uniform load, symmetrical concentrated load and mid-span concentrated load, the deformation of 1028 d is 1.017 × 10^−7^ mm, 3.44 × 10^−6^ mm and 1.88 × 10^−6^ mm, respectively. The deformation decreases according to the increase of stiffness, but the reduction is not significant. It shows that the influence of stiffness on member deformation is not obvious.

## 5. Conclusions

1. In this paper, the calculation formulas of interface slip, CFRP tension and steel beam deformation of steel beams strengthened with CFRP sheets under the combined action of temperature and creep are given by using the elastic method. The conclusions can be summarized as follows:
2. The finite element calculation results of the interface slip formula are compared with the formula results, and the results show that they are in good agreement. It shows that the formula is correct and can be used to calculate the interface slip between steel beam and CFRP sheet. The calculation formulas of CFRP tension and steel beam deformation are derived on the basis of the correctness of the formula.
3. The CFRP tension is distributed nonlinearly along the beam length, and the maximum is in the middle of the span and the minimum at the beam end tends to 0. CFRP sheet tension increases with the increase of connection stiffness. The greater the increase of connection stiffness, the smaller is the increase of CFRP sheet tension. When the stiffness reaches about 3 ka, the stiffness has little effect on the change of CFRP sheet tension. The tension of CFRP sheet increases with the increase of temperature (5–25 °C). The higher the temperature, the steeper is the distribution of the CFRP sheet tension curve. When the temperature is small, the distribution of the CFRP sheet tension curve is relatively flat. Temperature has a great influence on the tension of CFRP sheet. The tension of CFRP sheet increases with the increase of the thickness of CFRP sheet. The greater the thickness of CFRP sheet, the steeper is the distribution of CFRP cloth tension curve. When the thickness of CFRP sheet is small, the distribution of the tension curve of CFRP sheet is relatively flat. CFRP sheet tension has a nonlinear distribution with the increase of time. With increasing age, the tensile force of CFRP increases gradually. CFRP sheet tension increases with the increase of load. The greater the load, the steeper is the slip curve.
4. The deformation of steel beam decreases with the increase of stiffness, but the reduction range is very small. The effect of stiffness on reducing member deformation and improving its bearing capacity is not obvious. The deformation increases with the increase of temperature (5–25 °C), but the increase is not very large. The influence of temperature on the deformation and bearing capacity of members is not obvious. The deformation decreases with the increase of the thickness of CFRP sheet, but the reduction is not obvious. The change of CFRP sheet thickness has no obvious effect on the deformation and bearing capacity of members. With increasing age, the deformation increases gradually. The deformation increases with the increase of load. The greater the load, the steeper is the slip curve. When the load increases by 5 kN under creep, the deformation increases by about 2.2 × 10^−7^ mm, 1.8 × 10^−6^ mm, and 9.4 × 10^−7^ mm, respectively.
5. The theoretical derivation formula in this paper is based on a series of assumptions, and ignores the influence of some factors. Various factors should be taken into account in further research.

## Data Availability

Some of all data, models, of code that support the findings of this study are available from the corresponding author upon reasonable request.
